# Role of Circadian Rhythm Changes on Functional Dependence Despite Successful Repercussion in Patients with Endovascular Treatment

**DOI:** 10.2174/0115672026346635240816095721

**Published:** 2024-09-12

**Authors:** Mengke Zhang, Xian Wang, Xi Chen, Jiali Xu, Wenting Guo, Changhong Ren, Sijie Li, Wenbo Zhao, Chuanjie Wu, Xunming Ji

**Affiliations:** 1 Department of Neurology, Xuanwu Hospital, Capital Medical University, Beijing, China;; 2 Department of Health Medical Center, Sichuan Provincial People's Hospital, Sichuan, China;; 3 Department of Rehabilitation Medicine, Beijing ShiJiTan Hospital, Capital Medical University, Beijing, China;; 4 Department of Neurology, Zhejiang Provincial People’s Hospital, Zhejiang, China;; 5 Beijing Key Laboratory of Hypoxia Conditioning Translational Medicine, Xuanwu Hospital, Capital Medical University, Beijing, China;; 6 Emergency Department, Xuanwu Hospital, Capital Medical University, Beijing, China;; 7 Department of Neurosurgery, Xuanwu Hospital, Capital Medical University, Beijing, China;; 8 Laboratory of Brain Disorders, Laboratory for Clinical Medicine, Beijing Institute of Brain Disorders, Ministry of Science and Technology, Collaborative Innovation Center for Brain Disorders, Beijing Advanced Innovation Center for Big Data-based Precision Medicine, Capital Medical University, Beijing, China

**Keywords:** AIS, endovascular treatment, futile recanalization, circadian rhythm, outcome, LVO

## Abstract

**Background:**

Increasing evidence of circadian biology may influence the physiopathologic mechanism, progression, and recovery of stroke. However, few data have shown about circadian rhythm on futile recanalization (FR) in patients treated with endovascular treatment (EVT).

**Methods:**

From 2017 to 2021, an observational cohort of acute ischemic stroke (AIS) patients with large vessel occlusion (LVO) underwent EVT was conducted. FR was defined as the failure to achieve functional independence in patients at 90 days after EVT, although the occluded vessels reached a recanalization. The effect of circadian rhythm on FR was investigated using the logistic regression model.

**Results:**

Of 783 patients, there were 149 patients who had stroke onset between 23:00-6:59, 318 patients between 7:00-14:59, and 316 patients between 15:00-22:59. Patients suffered from stroke during 15:00-22:59 had shorter OTP (*p* =0.001) time, shorter OTR (*p*<0.001) time, higher rate of intravenous thrombolysis (*p* =0.001) than groups of other time intervals. The rate of FR post-EVT in patients who had a stroke between 15:00-22:59 was significantly higher than in those with stroke onset between 23:00-6:59 (*p* =0.017). After adjusting for confounding factors, the time of stroke occurring during 15:00-22:59 (adjusted OR [aOR], 1.652; 95%CI, 1.024-2.666, *p* =0.04) was an independent predictor of FR.

**Conclusion:**

Circadian rhythm can directly or indirectly affect the occurrence, development, and prognosis of AIS. More studies may be needed in the future to validate the results of our study and to explore the potential mechanisms behind the effects of circadian rhythms on FR.

## 1 INTRODUCTION

Acute Ischemic Stroke (AIS) due to large vessel occlusion (LVO) is associated with higher mortality and disability. The most effective treatment for LVO is rapid revascularization therapy. Patients with AIS who achieve recanalization post EVT and still have poor neurological function prognosis at 90 days are referred to as “futile recanalization (FR)” [1, 2]. The Reperfusion Efficacy Evaluation of multiple Stroke Endovascular Therapy Trials (HERMES) study suggested that one-third of patients achieved FR [3]. The results of RESCUE-RE showed that the proportion of FR is 49% in the real world in China [4].

So far, the mechanism of FR remains unclear, and exploring its predictive factors is of great value for early identification of poor prognosis after endovascular therapy. Previous studies have found a series of risk factors, mainly including old age, stroke severity, collateral circulation, and final infarct volume [2, 5, 6]. Increasing data have shown that clock genes and circadian rhythm have important effects on the occurrence, pathophysiological mechanism, progression, and recovery of AIS [7]. However, few studies have explored whether circadian rhythms also play a role in FR. Thus, the objective of our study was to assess the association between time of onset and the rate of FR in patients who underwent EVT.

## 2 METHODS

### 2.1 Study Design and Data Source

This study was conducted based on an observational cohort registered at Xuanwu Hospital, Capital Medical University. This prospective registry had been approved by the Ethics Committee. All patients or their legally authorized representatives were required to give both verbal and written consent upon admission. Consecutive AIS patients who underwent EVT were recruited in Xuanwu Hospital between January 2013 to June 2021. The inclusion criteria were 1) AIS secondary to LVO, 2) received modified treatment in cerebral ischemia (mTICI)≥2b/3a after recanalization treatment, and 3) available information of clinical data and function outcomes at 90 days.

### 2.2 Data Collection

The assessment data of patients included demographics, risk factors of cerebrovascular diseases, baseline National Institutes of Health Stroke Scale (NIHSS), Alberta Stroke Program Early CT Score (ASPECTS) or posterior circulation Alberta Stroke Program Early Computed Tomography Score (pc-ASPECTS), intravenous thrombolysis, time intervals, blood pressure, etiologies, symptomatic intracerebral hemorrhage (sICH), clinical functional outcomes at 3-month measured by modified Rankin Scale (mRS). Two experienced radiologists who were unaware of the clinical information of the patients evaluated the neuroimaging data independently. They were required to reach a consensus with regard to disagreement. Clinical outcomes were confirmed through standardized telephone interviews or clinical visits conducted by an investigator who was also unaware of the procedure. FR was defined as mRS > 2 scores at 90 days after successful vascular recanalization treatment (mTICI≥2b/3a). Patients were divided by binary time segmentations (23:00-06:59/ 07:00-22:59) and 8-h intervals (23:00-06:59/07:00-14:59/ 15:00-22:59) according to the recommendations of the Leducq Consortium International pour la Recherche Circadienne sur l’AVC Effects in Stroke (CIRCA) [8].

### 2.3 Statistical Analyses

All statistical analyses were conducted using IBM SPSS Statistics 27 (IBM Corp, Armonk, New York, USA), and significance was described as *p* < 0.05 (2-sided). According to the results of the normality test, continuous variables were displayed as mean±SD or median with interquartile range. Differences in continuous variables were confirmed using the Student t-test or Mann-Whitney U-test. Categorical variables were indicated as numbers (percentages). The chi-square test or Fisher exact test was applied for the comparisons between categorical data. The multivariable logistic regression was conducted to explore the role of the time of stroke onset in FR. The potential confounding factors (age, gender, hypertension, diabetes mellitus, hyperlipidemia, coronary heart disease, atrial fibrillation, ischemic stroke, intracranial hemorrhage, premorbid mRS, NIHSS, ASPECTS, treatment with IV alteplase, OTR time, SBP, DBP, posterior circulation infarct, sICH) were adjusted in this multivariable analysis based on the univariable logistic regression.

## 3 RESULTS

Of 906 AIS patients who received EVT, 850 patients achieved reperfusion after EVT, and 783 AIS patients eventually enrolled in this analysis. The flow chart of inclusion is demonstrated in Fig. (**1**).

While patients were categorized into groups based on the binary time segmentations, 634 patients had symptom onset in the daytime (7:00-22:59), and 149 patients had symptom onset in the nighttime (23:00-6:59). Patients during the daytime were likely to treat with IV alteplase (36.6% *vs.* 21.5%, *p* < 0.001), have shorter OTP time (370 *vs.* 450, *p < *0.001), OTR time (443 *vs.* 513, *p* < 0.001) compared with patients during nighttime (23:00-6:59) (Table **1**).

Based on the 8-h intervals, 149 patients had symptom onset during 23:00-6:59, 318 patients had symptom onset during 7:00-14:59, and 316 patients had symptom onset during 15:00-22:59. Patients with stroke onset during 15:00-22:59 were more likely to treat with IV alteplase when compared to other periods throughout the day (*p* =0.001). Compared with other time intervals, patients with short OTP (*p* =0.001) and OTR (*p* < 0.001) time tended to suffer from stroke during 15:00-22:59 (Table **2**).

**Fig. (2):**
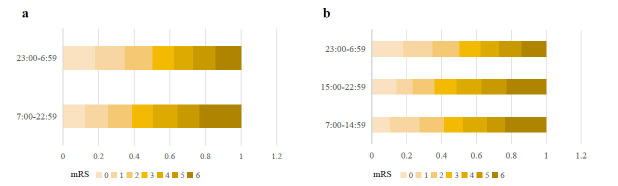
Clinical outcome after successful reperfusion at 3 months. (**a**) Distribution of modified Rankin Scale scores at 3 months post EVT in binary segmentations. (**b**) Distribution of modified Rankin Scale scores at 3 months post EVT in each 8-h interval.

**Fig. (3):**
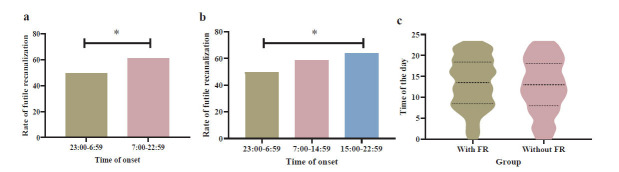
The rate of futile recanalization in different time segmentations. (**a**) Comparison between groups of binary segmentations. (**b**) Comparison between groups of 8-h intervals. (**c**) Time of onset between patients with and without FR. **Abbreviation:** FR= futile recanalization.

The rate of FR after EVT in patients with stroke onset during 23:00-6:59 was 49.66%, and 61.20% during 7:00-22:59 based on binary time segmentations. In terms of the segmentations of 8-h intervals, the rate of FR was 58.49% during 7:00-14:59, 63.92% during 15:00-22:59, and 49.66% during 23:00-6:59. (Fig. **2**) The patients with stroke onset in the 23:00-6:59 time were more likely to experience FR than those in 7:00-22:59 (*p* =0.01). FR was significantly associated with groups between the 15:00-22:59 time and 23:00-6:59 (*p* =0.017). In the violin plot, the distribution of onset time for patients with FR does not show a significant difference compared to that of patients without FR. However, the time-specificity of FR occurring between 0:00-5:00 time is slightly more obvious than that of effective reperfusion (Fig. **3**).

In the multivariable analysis, no significant difference was found between binary segmentations of time of stroke onset and FR after adjusting for other covariates in model 1. In the model, the time of stroke occurring during 15:00-22:59 (adjusted OR [aOR], 1.652; 95%CI [Confidence Interval], 1.024-2.666, *p* =0.04) was identified as an independent factor for predicting FR. And age (aOR, 1.668; 95%CI, 1.092-2.546, *p* =0.018), male (aOR, 1.039; 95%CI, 1.021-1.058, *p* < 0.001), hypertension (aOR, 0.661; 95%CI, 0.440-0.994, *p* =0.047), diabetes mellitus (aOR, 1.570; 95%CI, 1.052-2.343, *p* =0.027), hyperlipidemia (aOR, 2.818; 95%CI, 1.980-4.011, *p* < 0.001), NIHSS (aOR, 1.096; 95%CI, 1.069-1.122, *p* < 0.001), ASPECTS (aOR, 0.880; 95%CI, 0.785-0.986, *p* =0.027), DBP (aOR, 1.019; 95%CI, 1.002-1.035, *p* =0.025) were also related to FR (Table **3**).

## 4 DISCUSSION

In our study, we found that patients with stroke onset between 15:00-22:59 were more likely to achieve FR than those with stroke onset between 23:00-6:59. And in the multivariate logistic regression analysis, the time of stroke onset between 15:00-22:59 was the independent influence factor of FR after EVT.

Although FR is an important phenomenon that affects outcomes of patients, and its pathogenesis is not well understood. The status of collateral circulation and microvascular injury are relatively clear mechanisms affecting the FR [9]. Previous studies have shown that the common influencing factors for FR include old age, NIHSS at baseline, ASPECTS at baseline, anterior or posterior circulation, time of thrombectomy, and so on. The circadian rhythm is an intrinsic physiological cycle inherent to all living organisms that spans approximately 24 hours and regulates internal physiological functions in response to environmental changes. In mammals, circadian rhythms are governed by the biological clock [10]. While previous research observed the association between time of day and the clinical outcomes of AIS, it remains uncertain what the role of circadian rhythm is in FR. Most previous studies have shown that patients who had stroke onset at nighttime were more prone to have a poor prognosis. Wang *et al*. reported that patients with stroke occurring 00:00-06:00 and 06:00-12:00 tended to achieve favorable outcomes [11]. Burbano *et al*. suggested that morning treatment (05:00-10:59) was related to the lowest mRS scores at 90 days and successful recanalization [12]. A multicenter observational cohort study from Korea revealed that night-onset (18:00-22:00 and 22:00-02:00) stroke was more likely to achieve early neurological deterioration (END) and had a lower likelihood of favorable outcomes [13]. Similarly, another study found that patients disfavored EVT start times at 15:55-17:15 and 18:55-20:55 on working [14]. However, the time intervals of these studies are not uniform. In order to make the time division more standardized and rationalized, our research is in accordance with the latest consensus recommendations of the Leducq network on Circadian Effects in Stroke [8]. Consistent with most previous studies, we have further confirmed patients with stroke onset at the end of the day (15:00-22:59) benefit less from thrombectomy.

The occurrence of stroke follows a circadian rhythm, mainly related to the fluctuations of sympathetic nerve activity, blood pressure, coagulation factors, platelet aggregation, *etc*., and the circadian rhythms of these factors are controlled by endogenous clocks [15-17]. The potential mechanism of these results may be as follows. Firstly, previous experimental data in rats showed that the ischemic penumbra during the active phase in AIS was narrower than that during the resting period [18]. Accordingly, the active period of the human is the time of the day, which indicates that the cerebral infarction tissue saved by reperfusion during the day is less than that at night when AIS occurs. Secondly, preclinical data showed that the cerebral intravascular blood flow regulation had a circadian rhythm [7, 18]. Previous studies have shown that impaired automatic regulation of cerebral blood flow and venous outflow have been confirmed as possible mechanisms of FR [19, 20]. Therefore, using Transcranial Doppler (TCD) for continuous monitoring of cerebral autoregulation and CTA for venous outflow in the future will facilitate further investigation into the underlying mechanism through which circadian rhythm impacts reperfusion efficacy. Thirdly, circadian biology may impact the pathophysiology of BBB and edema following reperfusion, which may further lead to FR [7]. Additionally, body temperature between 15:00-22:59, the higher level of the day, perhaps results in a poor prognosis for patients after EVT, even if the vessel is successfully recanalization [21].

Some limitations should be considered. Firstly, our study was a single-center design, which may bring about the presence of selection bias. Secondly, imaging data were not included in our multivariate analysis because of the limited storage time of information about neuroimaging. Thirdly, some new indicators related to stroke circadian rhythm, such as vascular reactivity, are expected to be prospective studies in the future because relevant data have not been collected.

## CONCLUSION

There is accumulating evidence indicating that circadian rhythms influence stroke onset, pathophysiology, outcomes, and recovery in patients with AIS. Our study found the circadian rhythm that was associated with FR, and further research is warranted to explore the management of AIS patients who underwent EVT according to the circadian rhythm.

## Figures and Tables

**Fig. (1):**
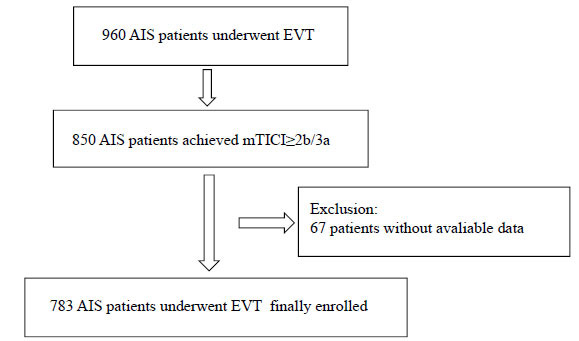
Flow chart. **Abbreviations:** AIS=acute ischemic stroke, EVT=endovascular treatment, mTICI=modified treatment in cerebral ischemia.

**Table 1:** Baseline characteristics in binary time segmentations.

**Variables**	**7:00-22:59 (n=634)**	**23:00-6:59 (n=149)**	***p* Value**
**Demographics**			
Age	63 (56-72)	64 (53-71)	0.175
Male	182 (28.7)	50 (33.6)	0.243
**Medical History**			
Hypertension	448 (70.7)	101 (67.8)	0.490
Diabetes mellitus	183 (28.9)	42 (28.2)	0.870
Hyperlipidemia	371 (58.5)	81 (54.4)	0.356
Coronary heart disease	136 (21.5)	24 (16.1)	0.145
Atrial fibrillation	216 (34.1)	42 (28.2)	0.169
Ischemic stroke	174 (27.4)	39 (26.2)	0.754
Intracranial hemorrhage	10 (1.6)	1 (0.7)	0.398
**Clinical Characteristics**			
Premorbid mRS	0 (0-0)	0 (0-0)	0.261
NIHSS	16 (12-22)	15 (11-21)	0.165
ASPECTS	9 (7-10)	9 (8-10)	0.439
Treatment with IV alteplase	232 (36.6)	32 (21.5)	<0.001
**Time Intervals**			
OTP time	370 (279-505)	450 (313-637)	<0.001
OTR time	443 (345.0-578.5)	513 (390.5-724.5)	<0.001
SBP	146 (131-164)	144 (130-160)	0.264
DBP	83 (75-91)	82 (76-90)	0.716
Posterior circulation infarct	184 (29.0)	40 (26.8)	0.597
TOAST	-	-	0.049
Large artery atherosclerosis	366 (57.7)	100 (67.1)	-
Cardioembolism	242 (38.2)	41 (27.5)	-
Other or undetermined	26 (4.1)	8 (5.4)	-
sICH	76 (12.0)	13 (8.7)	0.259

**Abbreviations:** NIHSS=National Institutes of Health Stroke Scale, ASPECTS=Alberta Stroke Program Early CT Score, IV= intravenous injection, OTP= time from onset to puncture, OTR=time from onset to reperfusion, SBP=systolic blood pressure, DBP= diastolic blood pressure, TOAST=etiology according to the Trial of Org 10172 in Acute Stroke Treatment, sICH= symptomatic intracerebral hemorrhage.

**Table 2:** Baseline characteristics in segmentations of 8-h intervals.

**Variables**	**23:00-6:59 (n=149)**	**7:00-14:59 (n=318)**	**15:00-22:59 (n=316)**	***p* Value**
**Demographics**				
Age	64 (53-71)	64 (55-72)	63 (56-71)	0.151
Male	50 (33.6)	92 (28.9)	90 (28.5)	0.502
**Medical History**				
Hypertension	101 (67.8)	217 (68.2)	231 (73.1)	0.322
Diabetes mellitus	42 (28.2)	90 (28.3)	93 (29.4)	0.939
Hyperlipidemia	81 (54.4)	176 (55.3)	195 (61.7)	0.175
Coronary heart disease	24 (16.1)	66 (20.8)	70 (22.2)	0.315
Atrial fibrillation	42 (28.2)	104 (32.7)	112 (35.4)	0.297
Ischemic stroke	39 (26.2)	85 (26.7)	89 (28.2)	0.877
Intracranial hemorrhage	1 (0.7)	3 (0.9)	7 (2.2)	0.277
**Clinical Characteristics**				
Premorbid mRS	0 (0-0)	0 (0-0)	0 (0-0)	0.474
NIHSS	15 (11-21)	16 (12-21)	17 (12-22)	0.337
ASPECTS	9 (8-10)	9 (7-10)	9 (7-10)	0.467
Treatment with IV alteplase	32 (21.5)	109 (34.3)	123 (38.9)	0.001
**Time Intervals**				
OTP time	450 (313-637)	380 (284.5-506.5)	362 (267.5-500.5)	0.001
OTR time	513 (390.5-724.5)	450 (343.5-570)	438.5 (345-586)	<0.001
SBP	144 (130-160)	147 (130-163)	146 (133-166)	0.418
DBP	82 (76-90)	83 (75-90)	83 (75-92)	0.862
Posterior circulation infarct	40 (26.8)	95 (29.9)	89 (28.2)	0.776
TOAST	-	-	-	0.140
Large artery atherosclerosis	100 (67.1)	179 (56.3)	187 (59.2)	-
Cardioembolism	41 (27.5)	124 (39.0)	118 (37.3)	-
Other or undetermined	8 (5.4)	15 (4.7)	11 (3.5)	-
sICH	13 (8.7)	38 (11.9)	38 (12.0)	0.528

**Abbreviations:** NIHSS=National Institutes of Health Stroke Scale, ASPECTS=Alberta Stroke Program Early CT Score, IV= intravenous injection, OTP= time from onset to puncture, OTR=time from onset to reperfusion, SBP=systolic blood pressure, DBP= diastolic blood pressure, TOAST=etiology according to the Trial of Org 10172 in Acute Stroke Treatment, sICH= symptomatic intracerebral hemorrhage.

**Table 3:** Multivariate analysis of between circadian rhythm and futile recanalization.

**Model 1**			**Model 2**		
**Variables**	**Adjusted OR (95% CI)**	***p* Value**	**Variables**	**Adjusted OR (95% CI)**	***p* Value**
Age	1.656 (1.085-2.527)	0.019	Age	1.668 (1.092-2.546)	0.018
Male	1.039 (1.021-1.058)	< 0.001	Male	1.039 (1.021-1.058)	< 0.001
Hypertension	0.669 (0.445-1.005)	0.053	Hypertension	0.661 (0.440-0.994)	0.047
Diabetes mellitus	1.573 (1.055-2.347)	0.026	Diabetes mellitus	1.570 (1.052-2.343)	0.027
Hyperlipidemia	2.831 (1.990-4.027)	< 0.001	Hyperlipidemia	2.818 (1.980-4.011)	< 0.001
Coronary heart disease	1.299 (0.813-2.074)	0.274	Coronary heart disease	1.306 (0.817-2.086)	0.265
Atrial fibrillation	0.950 (0.604-1.493)	0.823	Atrial fibrillation	0.936 (0.594-1.474)	0.775
Ischemic stroke	1.419 (0.915-2.200)	0.118	Ischemic stroke	1.422 (0.915-2.208)	0.117
Premorbid mRS	0.956 (0.688-1.327)	0.787	Premorbid mRS	0.957 (0.689-1.329)	0.791
NIHSS	1.096 (1.070-1.123)	< 0.001	NIHSS	1.096 (1.069-1.122)	< 0.001
ASPECTS	0.880 (0.785-0.986)	0.028	ASPECTS	0.880 (0.785-0.986)	0.027
Posterior circulation infarct	0.938 (0.598-1.471)	0.779	Posterior circulation infarct	0.946 (0.802-1.485)	0.809
SBP	1.006 (0.996-1.016)	0.223	SBP	1.006 (0.996-1.016)	0.223
DBP	1.019 (1.002-1.035)	0.026	DBP	1.019 (1.002-1.035)	0.025
sICH	11.101 (4.592-26.837)	< 0.001	sICH	11.048 (4.570-26.705)	< 0.001
Binary segmentations	-	-	8-h intervals	-	-
7:00-22:59	Reference	-	23:00-6:59	Reference	0.118
23:00-6:59	0.670 (0.433-1.037)	0.072	7:00-14:59	1.353 (0.843-2.173)	0.210
-	-	-	15:00-22:59	1.652 (1.024-2.666)	0.040

**Abbreviations:** NIHSS=National Institutes of Health Stroke Scale, ASPECTS=Alberta Stroke Program Early CT Score, SBP=systolic blood pressure, DBP= diastolic blood pressure, TOAST=etiology according to the Trial of Org 10172 in Acute Stroke Treatment, sICH= symptomatic intracerebral hemorrhage.

## Data Availability

The data and supportive information are available upon request from the corresponding author.

## LIST OF ABBREVIATIONS

AIS: Acute Ischemic Stroke
ASPECTS: Alberta Stroke Program Early CT Score
END: Early Neurological Deterioration
FR: Futile Recanalization
LVO: Large Vessel Occlusion
NIHSS: National Institutes of Health Stroke Scale
TCD: Transcranial Doppler

## STANDARDS OF REPORTING

STROBE guidelines were followed.
